# Supplementary material for: Chiral phonons in quartz probed by X-rays
Source: Nature. 2023 Jun 7;618(7967):946–50. doi: 10.1038/s41586-023-06016-5 (PMC10307621; doi:10.1038/s41586-023-06016-5)
Supplement: Supplementary file 1 — Supplementary Notes 1–5, Figs. 1–5, Tables 1–3 and References. [file 41586_2023_6016_MOESM1_ESM.pdf]

nature portfolio

https://doi.org/10.1038/s41586-023-06016-5

Supplementary information

Chiral phonons in quartz probed by X-rays

In the format provided by the
authors and unedited

nature portfolio

https://doi.org/10.1038/s41586-023-06016-5

Supplementary information

Chiral phonons in quartz probed by X-rays

In the format provided by the authors and unedited

Supplementary Information for "Chiral phonons probed by X rays"

Hiroki Ueda[1,2,*], Mirian García-Fernández[3], Stefano Agrestini[3], Carl P. Romao[4], Jeroen van den Brink[5,6], Nicola A. Spaldin[5], Ke-Jin Zhou[3], and Urs Staub[1,*]

[1] *Swiss Light Source, Paul Scherrer Institute, 5232 Villigen-PSI, Switzerland.*

[2] *SwissFEL, Paul Scherrer Institute, 5232 Villigen-PSI, Switzerland.*

[3] *Diamond Light Source, Harwell Campus, Didcot OX11 0DE, United Kingdom.*

[4] *Department of Materials, ETH Zurich, 8093 Zürich, Switzerland.*

[5] *Institute for Theoretical Solid State Physics, IFW Dresden, Helmholzstr. 20, 01069 Dresden, Germany.*

[6] *Institute for Theoretical Physics and Würzburg-Dresden Cluster of Excellence ct.qmat, Technische Universität Dresden, 01069 Dresden, Germany.*

[*] Correspondence authors: hiroki.ueda@psi.ch and urs.staub@psi.ch

SUPPLEMENTARY NOTE 1: *Transfer of angular momentum to phonons in RIXS*

The initial and the final states of the RIXS process are $|0\rangle \otimes |\mathbf{Q}, \boldsymbol{\epsilon}\rangle$ and $|m\rangle \otimes |\mathbf{Q}', \boldsymbol{\epsilon}'\rangle$, respectively, where $|0\rangle$ is the ground state of the material described by Hamiltonian H_0, and $|m\rangle$ is a generic eigenstate of this Hamiltonian with a certain number of phonons with energy E_m with respect to $|0\rangle$, while $\mathbf{Q}$ ($\mathbf{Q}'$) is the momentum and $\boldsymbol{\epsilon}$ ($\boldsymbol{\epsilon}'$) is the polarization of the incident (scattered) photon. During the RIXS process, a momentum $\mathbf{q} \equiv \mathbf{Q} - \mathbf{Q}'$ and an energy $\omega = E_m$ are transferred from the photon to the system.

To capture how in RIXS angular momentum can be transferred to phonons, we consider an elemental building block of a solid in which an atom can rotate around a global z-axis at a fixed distance to that axis. This simplifies the zero-temperature RIXS amplitude as it is now independent of $\mathbf{q}$. At the O K edge, the RIXS intensity is $I(\omega) = \sum_m |A_m|^2 \delta(\omega - E_m)$, where A_m is the scattering amplitude from $|0\rangle \otimes |\mathbf{Q}, \boldsymbol{\epsilon}\rangle$ to $|m\rangle \otimes |\mathbf{Q}', \boldsymbol{\epsilon}'\rangle$. This amplitude is given by the Kramers-Heisenberg formula:

$$A_m = \sum_n \frac{\langle m|D_{\boldsymbol{\epsilon}'}^\dagger|n\rangle\langle n|D_{\boldsymbol{\epsilon}}^\dagger|0\rangle}{\Omega - \mathcal{E}_n + i\Gamma} = \sum_n \langle m|D_{\boldsymbol{\epsilon}'}^\dagger|n\rangle G_c \langle n|D_{\boldsymbol{\epsilon}}^\dagger|0\rangle, \qquad (1)$$

where the sum is over intermediate states $|n\rangle$ with energy $\mathcal{E}_n$ of the intermediate state Hamiltonian $H_0 + H_I$, where the interaction between the intermediate state exciton and the rest of the system is given by H_I. Γ is the inverse lifetime of the core-hole and Ω is the energy of the incident photon with respect to the resonance energy (i.e., the energy difference between the $1s$ and $2p$ shells), and the operator $D_{\boldsymbol{\epsilon}}^\dagger$ promotes a $1s$ electron into a $2p$ state while at the same time the incident photon with polarization $\boldsymbol{\epsilon}$ is annihilated. In the last line, the intermediate state propagator $G_c(\Omega) = (\Omega - H_0 - H_I + i\Gamma)^{-1}$ is defined [1].

So to establish a theoretical model for a RIXS process in general, three essential building blocks are required: the Hamiltonian H_0 of the system in the initial and final state without the core-hole, the interaction Hamiltonian H_I of the intermediate state core-hole – valence electron exciton with the rest of the system, and the dipole operators $D_{\boldsymbol{\epsilon}}$ and $D_{\boldsymbol{\epsilon}'}$ creating/annihilating the intermediate state exciton, respectively, that are functions of the incoming x-ray polarization $\boldsymbol{\epsilon}$ and outgoing one $\boldsymbol{\epsilon}'$.

We consider the atom to have a local electronic basis of two p-orbitals p_x and p_y (that are, in general, not degenerate) plus an s core orbital. Depending on the angle ϕ, in the local frame of the atom, a p_x-orbital or p_y (or a linear combination of both) is pointing towards the center of rotation, see Fig. S1. To construct H_0, we consider that before the RIXS process the s-shell is filled and p-shell is empty, and the kinetic part of the Hamiltonian is given by the rotor $\frac{L_z^2}{2I}$, where I is the moment of inertia, L_z is the angular momentum operator $L_z = -i\partial_\phi$, and the eigenstates are $|l\rangle = \exp(il\phi)$. In addition, a potential of the form $v\cos\phi$ localizes

the atom around $\phi = 0$ (when $v < 0$), the equilibrium point around which it can oscillate. So the Hamiltonian of the system without core-hole is

$$H_0 = \frac{{L_z}^2}{2I} + v\cos\phi, \quad (2)$$

which is the Hamiltonian of the quantum pendulum [2], with eigenstates for the phonons $|m\rangle = \sum_l v_l^m |l\rangle$ that correspond to Mathieu functions. The ground state expectation value for the angular momentum is $\langle L_z\rangle_0 = \hbar\langle 0|L_z|0\rangle = \hbar\sum_m m|v_m^0|^2$. It is instructive to consider H_0 for large I, when an expansion of ϕ at the minimum of the potential reduces the Hamiltonian to a harmonic oscillator, as expected for local phonon excitations. A difference with the quantum harmonic oscillator is still in the boundary conditions, which are periodic in ϕ for the pendulum.

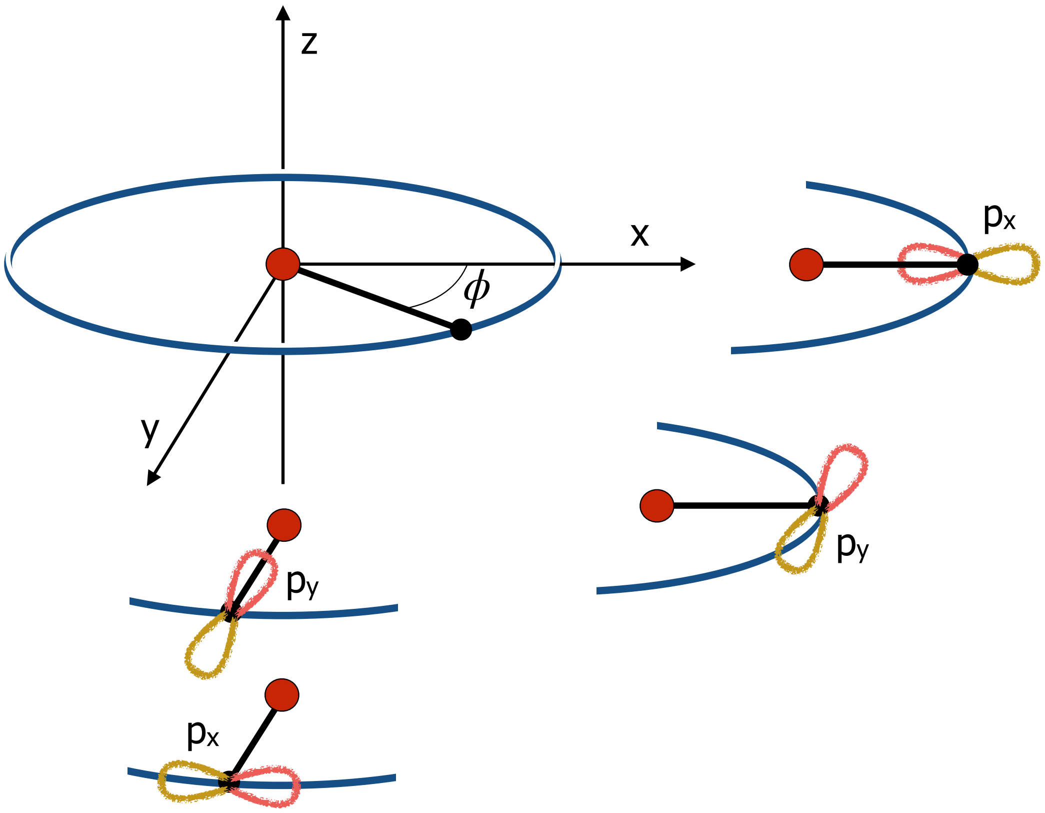

Fig. S1 | Definition of x, y, z-axis, angle ϕ, p_x and p_y-orbital. As the atom rotates a different linear combination of p_x and p_y orbitals are pointing towards the z-axis.

We now need to establish how the quantum pendulum couples to the electronic degree of freedom, i.e., define how in the RIXS intermediate state, the core-hole s – valence-shell p exciton couples to the pendulum. The creation operator for an electron in the orbital pointing towards the center of rotation for given ϕ is $p_{\phi+}^\dagger = p_x^\dagger \cos\phi + p_y^\dagger \sin\phi$ and its

orthogonal counterpart, pointing along the tangential direction is $p_{\phi-}^{\dagger} = -p_x^{\dagger}\sin\phi + p_y^{\dagger}\cos\phi$. We construct H_I such that it has $p_{\phi+}^{\dagger}|0\rangle$ as its ground state for any ϕ: consequently during an (adiabatically slow) rotation of the atom around the z-axis, the ground state wavefunction keeps pointing towards the axis of rotation. At the same time, the wavefunction of the excited state at energy 2α is $p_{\phi-}^{\dagger}|0\rangle$. This leads to the interaction Hamiltonian H_I for the RIXS intermediate state

$$H_I = \alpha s s^{\dagger}\left(p_{\phi-}^{\dagger}p_{\phi-} - p_{\phi+}^{\dagger}p_{\phi+}\right) = -\alpha s s^{\dagger}\mathbf{p}^{\dagger}(\sigma_z\cos 2\phi + \sigma_x\sin 2\phi)\mathbf{p}, \quad (3)$$

where $ss^{\dagger}$ is the core-hole density operator, we defined the vector operator $\mathbf{p} = \left(p_x, p_y\right)$ and σ_i denotes the Pauli matrixes with $i = x, y, z$. This Hamiltonian exactly fulfils the conditions under rotation required above. The dipole operator associated to an incident photon with polarization $\boldsymbol{\epsilon} = \left(\epsilon_x, \epsilon_y\right)$ perpendicular to the z-axis creates an s core-hole while promoting an electron to the p-shell: $D_{\boldsymbol{\epsilon}} = \boldsymbol{\epsilon}\cdot\mathbf{p}^{\dagger}s + h.c.$ and similarity for a scattered photon with polarization $\boldsymbol{\epsilon}'$.

Photon excitations are created via the indirect RIXS process, i.e., phonons cannot be excited via the dipole matrix elements directly but their creation in the RIXS process crucially depends on H_I. In this situation, the ultrashort core-hole lifetime (UCL) approximation can be used to determine the RIXS scattering operator, which formally holds when the electron-phonon coupling constant $\alpha^2 \ll \Omega^2 + \Gamma^2$. The sum over intermediate states in Eq. (1) can be avoided and the expression for the RIXS amplitude reduces to

$$A_m^{UCL} \propto \langle m|D_{\boldsymbol{\epsilon}'}^{\dagger}H_I D_{\boldsymbol{\epsilon}}^{\dagger}|0\rangle \propto (\boldsymbol{\epsilon}')^{*}\langle m|\sigma_z\cos 2\phi + \sigma_x\sin 2\phi\,|0\rangle\boldsymbol{\epsilon}. \quad (4)$$

Introducing the circular polarization basis $\boldsymbol{\epsilon}_c = \left(\epsilon_x + i\epsilon_y, \epsilon_x - i\epsilon_y\right)$, where a fully left circularly polarized photon corresponds to $\boldsymbol{\epsilon}_c^L = (1, 0)$ and a right one to $\boldsymbol{\epsilon}_c^R = (0, 1)$, the RIXS amplitude becomes

$$A_m^{UCL} \propto \langle m|(\boldsymbol{\epsilon}_c')^{*}\begin{pmatrix} 0 & e^{-2i\phi} \\ e^{2i\phi} & 0 \end{pmatrix}\boldsymbol{\epsilon}_c|0\rangle. \quad (5)$$

Thus, if the circular polarization of the photon in the scattering process changes from left to right or vice versa, the scattering operator for the system is $e^{\pm 2i\phi}$. From Eq. (2), it follows that the scattering matrix element $\langle m|e^{\pm 2i\phi}|0\rangle = \langle m|(L^{\pm})^2|0\rangle$ causes a phononic excitation in the system with an angular momentum expectation value $\Delta\langle L_z\rangle = \langle L_z\rangle_m - \langle L_z\rangle_0 = \pm 2\hbar$. Therefore, during the RIXS process, an angular momentum of up to $2\hbar$ may be transferred to the local phonon excitations that we consider here, depending on the precise (circular) polarization that the incoming and scattered photons have.

SUPPLEMENTARY NOTE 2: *RIXS spectra at the other **Q** points*

In this section, we present RIXS spectra obtained at different momentum points than $\mathbf{Q}_1$ = (-0.25, 0, 0.32), i.e., $\mathbf{Q}_2$ = (-0.29, 0.14, 0.32) and $\mathbf{Q}_3$ = (-0.25, 0.25, 0.32). Because of phonon dispersion, RIXS spectra are different at the three momentum points, $\mathbf{Q}_1$, $\mathbf{Q}_2$, and $\mathbf{Q}_3$.

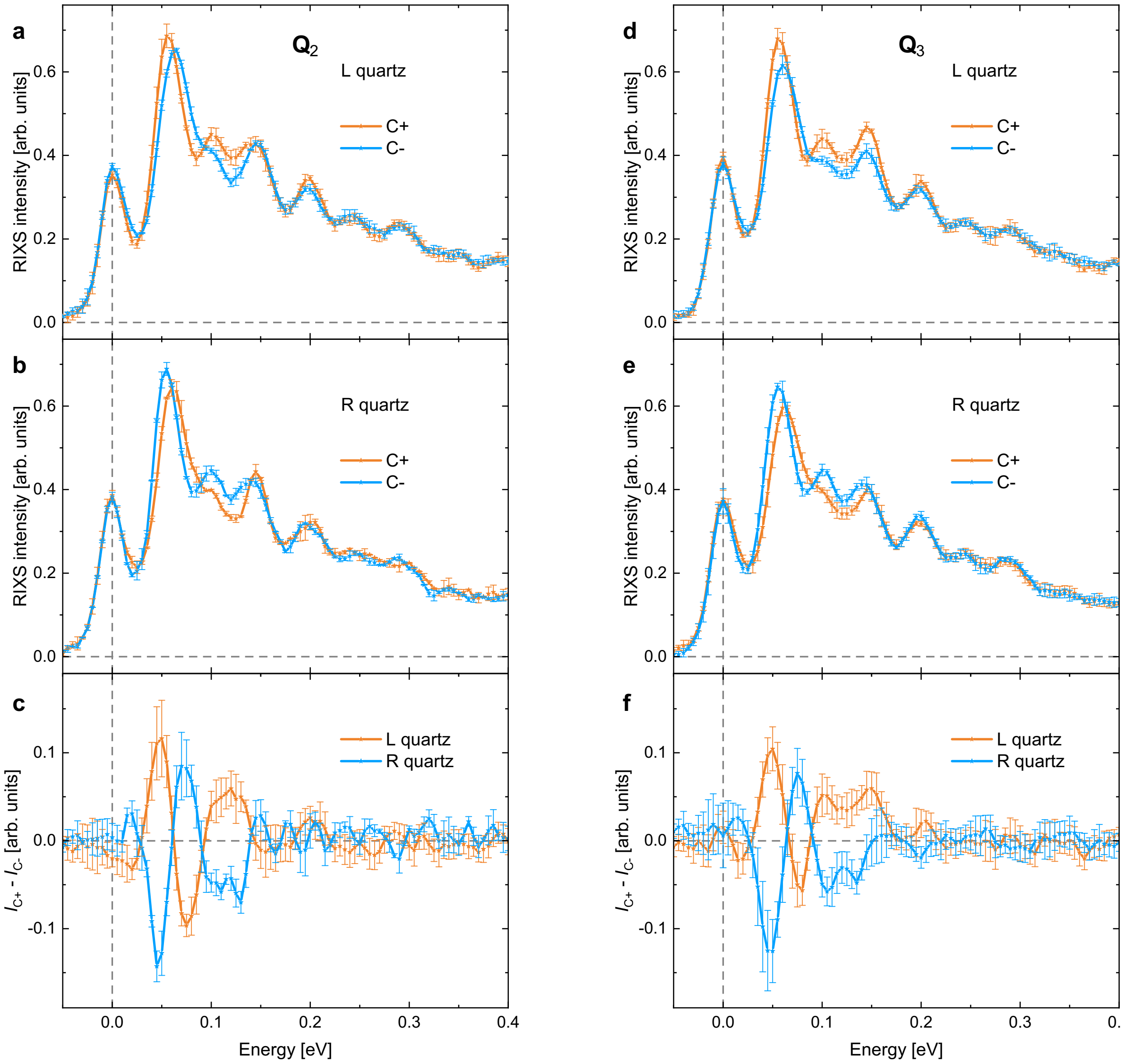

Fig. S2 | RIXS with circularly-polarized X rays obtained at different momentum points. Comparison between **a** [**d**], left quartz and **b** [**e**], right quartz, taken at the incident photon energy of 534 eV and at $\mathbf{Q}_2$ = (-0.29, 0.14, 0.32) [$\mathbf{Q}_3$ = (-0.25, 0.25, 0.32)], and **c** [**f**], extracted circular dichroic components of the data from **a** and **b** [**d** and **e**].

SUPPLEMENTARY NOTE 3: *Non-chiral rotational phonon mode*

The upper part of Fig. S3 represents a simple schematic picture of a rotational phonon mode that propagates in the rotation plane. Applying the two-fold rotation operation (C_2) switches the rotation direction while the propagation is intact, meaning that two phonon

modes with the opposite rotation direction are connected without a space-inversion operation or a mirror operation. Thus, a rotational phonon mode whose propagation direction lies in the rotation plane does not have a chiral character.

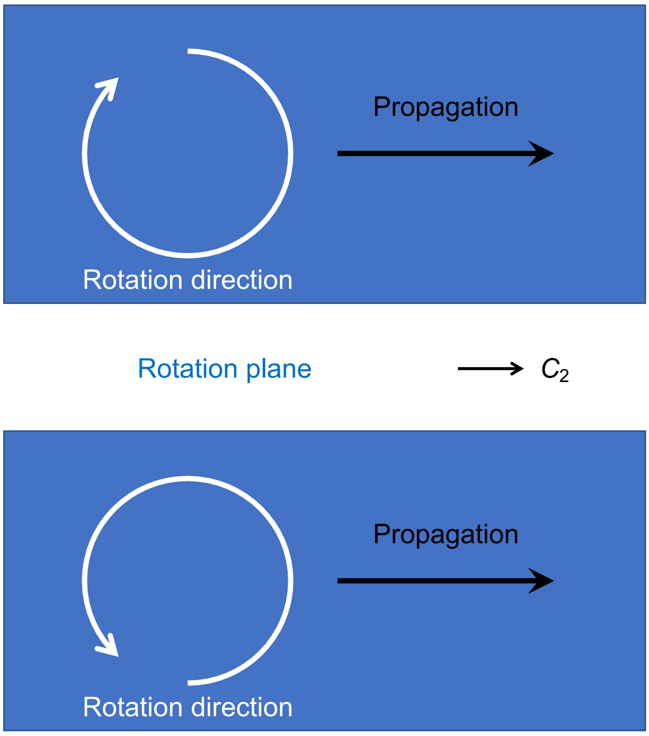

Fig. S3 | Non-chiral nature of a rotational phonon mode propagating in the rotation plane. A rotational mode that propagates to the right side (up) is superimposed on the other mode with the opposite rotation direction (down) by the two-fold rotation operation.

SUPPLEMENTARY NOTE 4: *Additional phonon band structures*

Figures S4 and S5 show the calculated phonon circular polarization and magnetic moments of quartz, respectively, throughout the Brillouin zone, including at all measured **Q**-points [$\mathbf{Q}_1$ = (−0.25, 0, 0.32), $\mathbf{Q}_2$ = (−0.29, 0.14, 0.32), $\mathbf{Q}_3$ = (−0.22, 0.25, 0.32)]. Quartz is shown to possess a high degree of phonon chirality, as most bands except the Si–O stretches above 100 meV carry a significant circular polarization. However, many highly circularly polarized modes do not carry relatively large magnetic moments, as there is a further restriction for the magnetic moments of the individual sublattices not to cancel.

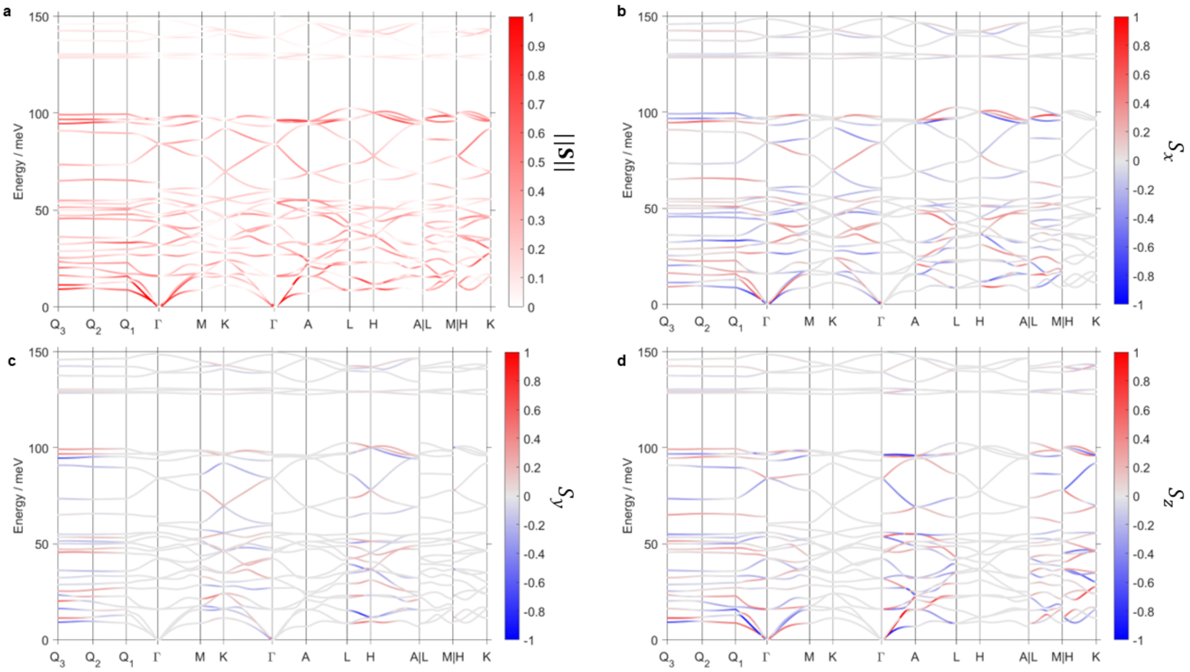

Fig. S4 | Phonon band structure of quartz coloured according to the magnitude (||S||). (**a**) and *x*, *y*, and *z* components (**b**–**d**) of the phonon circular polarization vector (**S**), for the measured **Q**-points and the high symmetry points in the Brillouin zone.

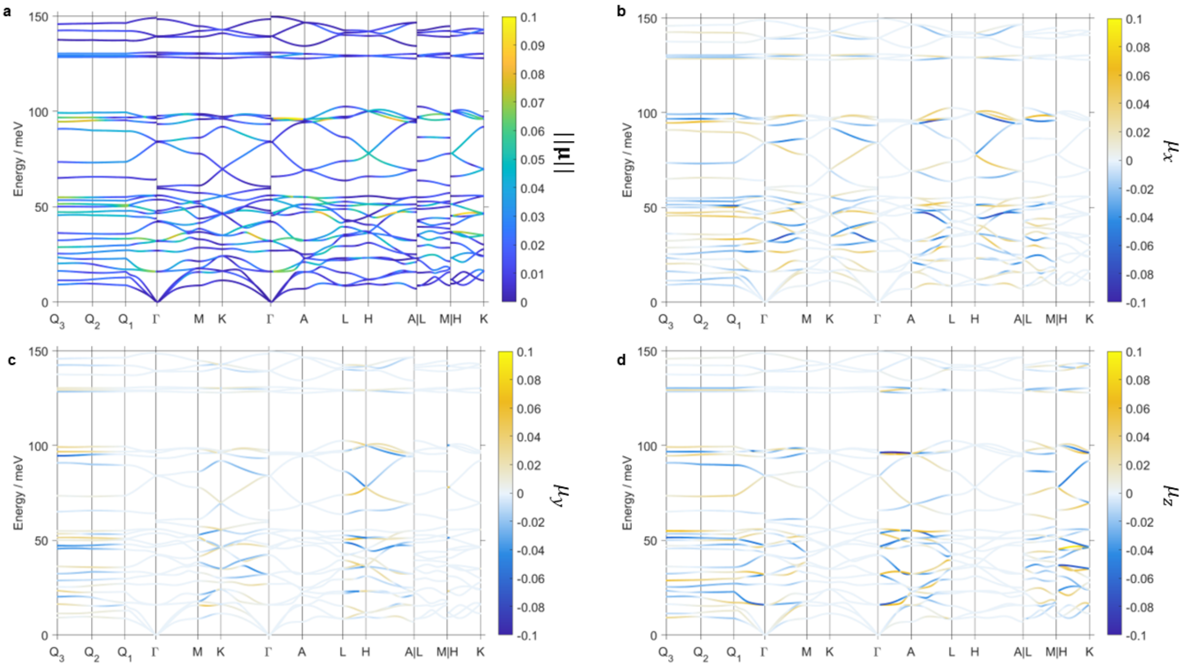

Fig. S5 | Phonon band structure of quartz coloured according to the magnitude (||μ||). (**a**) and *x*, *y*, and *z* components (**b**–**d**) of the phonon magnetic moment (**μ**, given in units of the

nuclear magneton), for the measured **Q-**points and the high symmetry points in the Brillouin zone.

SUPPLEMENTARY NOTE 5: *Calculated phonon circular polarizations*

In the following tables we present the DFT-calculated energy (*E*) and circular polarization (**S**) of all phonon bands at the three measured **Q**-points [$\mathbf{Q}_1$ = (−0.25 0 0.32), $\mathbf{Q}_2$ = (−0.29 0.14 0.32), $\mathbf{Q}_3$ = (−0.25 0.25 0.32)] as well as the circular polarization resolved into the contributions of the Si and O sublattices ($\mathbf{S}_{Si}$, $\mathbf{S}_{O}$).

SUPPLEMENTARY *Tables*

Table S1: $\mathbf{Q}_1$

E /meV	S_x	S_y	S_z	$\|\|\mathbf{S}\|\|$	$S_{x,\mathrm{Si}}$	$S_{y,\mathrm{Si}}$	$S_{z,\mathrm{Si}}$	$\|\|\mathbf{S}_{\mathrm{Si}}\|\|$	$S_{x,\mathrm{O}}$	$S_{y,\mathrm{O}}$	$S_{z,\mathrm{O}}$	$\|\|\mathbf{S}_{\mathrm{O}}\|\|$
8.491	-0.328	0.000	0.432	0.533	-0.1764	0	0.1506	0.232	-0.1467	0	0.2738	0.311
12.679	-0.074	0.000	-0.057	0.091	-0.0318	0	0.0028	0.032	-0.0486	0	-0.0457	0.067
15.823	0.301	0.000	-0.578	0.647	0.1768	0	-0.2555	0.311	0.1236	0	-0.317	0.340
17.228	0.224	0.000	-0.147	0.264	0.0519	0	0.0273	0.059	0.1658	0	-0.176	0.242
22.751	0.249	0.000	0.302	0.396	0.088	0	0.0536	0.103	0.1711	0	0.2457	0.299
27.014	0.065	0.000	0.084	0.096	-0.0421	0	-0.0153	0.045	0.1036	0	0.0884	0.136
29.704	0.203	0.000	-0.080	0.219	0.0541	0	0.0004	0.054	0.1487	0	-0.0832	0.170
33.246	-0.623	0.000	-0.047	0.624	-0.1722	0	0.0145	0.173	-0.451	0	-0.0537	0.454
35.723	-0.014	0.000	0.020	0.023	0.0056	0	-0.0127	0.014	-0.016	0	0.0334	0.037
45.112	-0.229	0.000	0.086	0.255	0.0557	0	0.0335	0.065	-0.2955	0	0.0533	0.300
47.596	-0.224	0.000	0.303	0.364	-0.0895	0	0.0646	0.110	-0.1196	0	0.2329	0.262
50.154	-0.211	0.000	-0.311	0.378	0.1099	0	-0.0752	0.133	-0.3259	0	-0.2356	0.402
50.907	0.349	0.000	0.076	0.355	-0.0186	0	-0.0205	0.028	0.3669	0	0.0874	0.377
53.126	0.067	0.000	-0.148	0.154	-0.0701	0	-0.0367	0.079	0.1393	0	-0.1012	0.172
55.022	0.029	0.000	-0.033	0.042	-0.0957	0	-0.022	0.098	0.1259	0	-0.0071	0.126
65.373	0.326	0.000	0.133	0.350	0.2619	0	0.0486	0.266	0.0616	0	0.0849	0.105
73.427	-0.075	0.000	0.022	0.076	-0.1238	0	0.1728	0.213	0.0505	0	-0.1517	0.160
89.573	-0.012	0.000	-0.270	0.271	0.0655	0	-0.3151	0.322	-0.0835	0	0.0445	0.095
95.446	0.086	0.000	-0.002	0.096	0.0692	0	0.0048	0.069	0.0264	0	-0.0011	0.026
96.827	0.203	0.000	0.105	0.216	0.1766	0	0.0856	0.196	0.0116	0	0.0194	0.023
99.518	-0.342	0.000	0.150	0.363	-0.3101	0	0.1276	0.335	-0.0237	0	0.016	0.029
128.520	0.083	0.000	-0.033	0.075	0.1485	0	-0.0341	0.152	-0.0777	0	0.0102	0.078
129.043	-0.080	0.000	0.084	0.098	-0.1294	0	0.1281	0.182	0.0584	0	-0.0606	0.084
130.396	-0.060	0.000	-0.087	0.100	-0.105	0	-0.1425	0.177	0.0483	0	0.0599	0.077
137.354	0.029	0.000	-0.057	0.064	0.0155	0	-0.0368	0.040	0.0142	0	-0.0196	0.024
142.082	0.083	0.000	0.031	0.095	0.1027	0	0.0112	0.103	-0.0121	0	0.0184	0.022
146.565	-0.026	0.000	0.023	0.041	-0.0173	0	0.0405	0.044	-0.0156	0	-0.0157	0.022

Table S2: $\mathbf{Q}_2$

E /meV	S_x	S_y	S_z	$\|\|\mathbf{S}\|\|$	$S_{x,\mathrm{Si}}$	$S_{y,\mathrm{Si}}$	$S_{z,\mathrm{Si}}$	$\|\|\mathbf{S}_{\mathrm{Si}}\|\|$	$S_{x,\mathrm{O}}$	$S_{y,\mathrm{O}}$	$S_{z,\mathrm{O}}$	$\|\|\mathbf{S}_{\mathrm{O}}\|\|$
9.433	-0.092	-0.162	0.025	0.188	-0.0799	-0.0484	0.012	0.094	-0.0247	-0.0892	0.0215	0.095
11.632	-0.032	0.015	0.063	0.072	-0.0318	0.0112	0.0475	0.058	0.0071	-0.0196	0.0054	0.022
15.384	0.106	-0.004	-0.084	0.135	0.06	0.01	-0.0223	0.065	0.0612	-0.0046	-0.0585	0.085
20.001	0.047	-0.094	-0.103	0.147	0.0293	-0.0694	-0.0609	0.097	0.0094	-0.0356	-0.0497	0.062
22.555	0.246	0.151	0.079	0.299	0.1106	0.0744	0.0131	0.134	0.136	0.071	0.0714	0.169
26.014	0.026	0.222	0.031	0.225	-0.0356	0.1265	-0.0589	0.144	0.0533	0.1046	0.0926	0.150
29.023	0.073	-0.044	-0.051	0.100	0.0278	-0.045	0.0568	0.078	0.0462	-0.0036	-0.1115	0.121
33.312	-0.271	-0.050	0.043	0.278	-0.1045	-0.0735	0.0296	0.131	-0.1597	0.0255	0.0162	0.163
35.806	-0.068	-0.168	-0.112	0.213	-0.0017	-0.0079	-0.0512	0.052	-0.0665	-0.1582	-0.0606	0.182
45.421	-0.230	0.269	0.078	0.362	0.0067	0.0375	0.0022	0.038	-0.2475	0.2234	0.0696	0.341
47.189	-0.203	0.113	0.166	0.285	0.0313	-0.0689	0.0337	0.083	-0.216	0.1884	0.1188	0.310
49.874	0.031	-0.093	-0.105	0.144	-0.0383	0.0267	0.0234	0.052	0.0718	-0.1288	-0.1148	0.187
51.205	0.063	-0.164	0.121	0.213	0.0107	-0.0038	-0.0462	0.048	0.0442	-0.1593	0.1517	0.224
53.354	0.077	0.148	-0.042	0.172	-0.0838	0.0755	-0.085	0.141	0.1535	0.0758	0.06	0.181
54.846	0.064	-0.065	-0.201	0.221	-0.0176	0.0167	0.0222	0.033	0.0882	-0.0764	-0.217	0.246
65.687	0.235	-0.008	0.228	0.327	0.2135	-0.045	0.1307	0.254	0.0185	0.0342	0.0971	0.105
73.081	-0.069	-0.026	-0.141	0.159	-0.1407	0.0268	0.0248	0.145	0.0684	-0.0537	-0.1665	0.188
89.935	0.030	-0.178	-0.185	0.258	0.0936	-0.1729	-0.2327	0.305	-0.0636	0.0012	0.043	0.077
95.252	0.543	-0.236	0.312	0.669	0.5322	-0.228	0.2987	0.651	0.0174	-0.0022	0.0138	0.022
96.793	-0.381	0.177	-0.331	0.534	-0.3898	0.1862	-0.3254	0.541	0.0091	-0.0199	0.0074	0.023
99.446	-0.227	0.225	0.242	0.401	-0.2105	0.1941	0.2158	0.359	-0.0191	0.0299	0.0159	0.039
128.487	0.070	-0.043	-0.042	0.093	0.1293	-0.0828	-0.0522	0.162	-0.0668	0.0383	0.0165	0.079
129.127	-0.069	0.029	0.083	0.111	-0.1126	0.0566	0.1344	0.184	0.0512	-0.0278	-0.0629	0.086
130.345	-0.051	0.031	-0.083	0.102	-0.0926	0.0562	-0.1417	0.178	0.0419	-0.0229	0.0597	0.076
137.526	0.031	-0.008	-0.049	0.058	0.0157	0.0014	-0.0247	0.029	0.013	-0.0089	-0.0217	0.027
142.167	0.082	-0.052	0.046	0.108	0.1008	-0.058	0.0207	0.118	-0.0106	0.0043	0.0241	0.027
146.274	-0.032	0.018	0.012	0.038	-0.022	0.0039	0.0353	0.042	-0.0159	0.0143	-0.0216	0.030

Table S3: $\mathbf{Q}_3$

E /meV	S_x	S_y	S_z	$\|\|\mathbf{S}\|\|$	$S_{x,\mathrm{Si}}$	$S_{y,\mathrm{Si}}$	$S_{z,\mathrm{Si}}$	$\|\|\mathbf{S}_{\mathrm{Si}}\|\|$	$S_{x,\mathrm{O}}$	$S_{y,\mathrm{O}}$	$S_{z,\mathrm{O}}$	$\|\|\mathbf{S}_{\mathrm{O}}\|\|$
8.943	0.255	-0.442	-0.547	0.748	0.098	-0.1698	-0.1789	0.265	0.1553	-0.269	-0.3758	0.488
11.331	-0.229	0.396	0.567	0.728	-0.1015	0.1758	0.2379	0.313	-0.1262	0.2187	0.333	0.418
16.354	0.199	-0.345	0.093	0.409	0.0554	-0.0959	0.0102	0.111	0.15	-0.2598	0.0795	0.310
20.162	-0.265	0.459	-0.105	0.541	-0.0802	0.1389	-0.0798	0.179	-0.1897	0.3286	-0.0235	0.380
23.451	0.173	-0.299	0.219	0.408	0.0453	-0.0784	0.0585	0.108	0.1251	-0.2167	0.1528	0.293
25.404	-0.136	0.236	-0.197	0.337	-0.063	0.1091	-0.1292	0.180	-0.0742	0.1285	-0.0572	0.159
28.752	0.032	-0.055	-0.099	0.118	0.0304	-0.0527	0.0855	0.105	0.0023	-0.0039	-0.1878	0.188
32.339	0.013	-0.023	0.116	0.119	0.0332	-0.0574	0.0338	0.074	-0.0182	0.0315	0.0904	0.097
36.140	0.049	-0.084	-0.201	0.224	-0.0053	0.0092	-0.0776	0.078	0.0525	-0.091	-0.1245	0.163
45.429	-0.203	0.352	0.028	0.407	-0.0317	0.0549	-0.0475	0.079	-0.1675	0.2901	0.0596	0.340
47.118	-0.093	0.161	0.097	0.209	0.0587	-0.1017	0.065	0.134	-0.1515	0.2624	0.0267	0.304
49.982	0.027	-0.047	0.050	0.074	-0.0219	0.0379	0.0426	0.061	0.0513	-0.0889	0.0242	0.105
51.345	0.066	-0.114	0.293	0.321	-0.006	0.0104	-0.0365	0.038	0.0712	-0.1233	0.3256	0.355
53.469	0.012	-0.021	-0.071	0.075	-0.0576	0.0998	-0.1441	0.185	0.0609	-0.1055	0.078	0.145
54.885	0.031	-0.053	-0.326	0.332	-0.014	0.0242	0.0864	0.091	0.0477	-0.0826	-0.4095	0.420
65.087	0.000	0.000	0.286	0.286	0.0294	-0.051	0.1996	0.208	-0.0278	0.0482	0.0895	0.105
73.473	0.059	-0.102	-0.281	0.304	0.0083	-0.0144	-0.1183	0.119	0.0506	-0.0876	-0.1616	0.191
90.709	0.083	-0.144	0.022	0.167	0.085	-0.1473	-0.0433	0.175	-0.0031	0.0054	0.0677	0.068
94.559	0.304	-0.527	0.087	0.615	0.3022	-0.5235	0.0875	0.611	-0.0014	0.0024	-0.0097	0.010
96.824	-0.220	0.382	-0.251	0.507	-0.2182	0.3779	-0.2569	0.506	0.0027	-0.0047	0.007	0.009
99.263	-0.181	0.313	0.244	0.436	-0.1664	0.2882	0.2277	0.403	-0.0135	0.0234	0.0197	0.033
128.435	0.044	-0.075	-0.049	0.100	0.0814	-0.141	-0.0653	0.175	-0.039	0.0676	0.0208	0.081
129.096	-0.031	0.054	0.082	0.103	-0.0575	0.0996	0.1353	0.178	0.0275	-0.0477	-0.0626	0.083
130.434	-0.032	0.055	-0.082	0.104	-0.0557	0.0964	-0.14	0.179	0.0235	-0.0408	0.059	0.075
137.633	0.013	-0.023	-0.038	0.047	0.005	-0.0086	-0.0141	0.017	0.0084	-0.0146	-0.0238	0.029
142.224	0.041	-0.071	0.061	0.102	0.0445	-0.0771	0.0312	0.094	-0.0025	0.0043	0.033	0.033
146.124	-0.011	0.019	0.001	0.022	0.0021	-0.0036	0.0306	0.031	-0.0144	0.025	-0.0305	0.042

SUPPLEMENTARY *Movies*

Movie 1 Supplementary Movie 1 from figure 5c visualizing mode X at $\mathbf{Q}_1$, and shows that it involves a circular motion of the atoms.

Movie 2 Supplementary Movie 2 visualize the evolution of the local charge quadrupoles at the O site when the chiral phonon mode is excited.

SUPPLEMENTARY *References*

1. Ament, L. P. J., Forte, F., and van den Brink, J. Ultrashort lifetime expansion for indirect resonant inelastic x-ray scattering. *Phys. Rev. B* **75**, 115118 (2007).
2. Baker, G. L., Blackburn, J. A., & Smith, H. J. T. The quantum pendulum: Small and large. *Am. J. Phys.* **70**, 525-531 (2002).
