# Supplementary figures and images for: Chiral phonons in quartz probed by X-rays
Source: Nature. 2023 Jun 7;618(7967):946–50. doi: 10.1038/s41586-023-06016-5 (PMC10307621; doi:10.1038/s41586-023-06016-5)

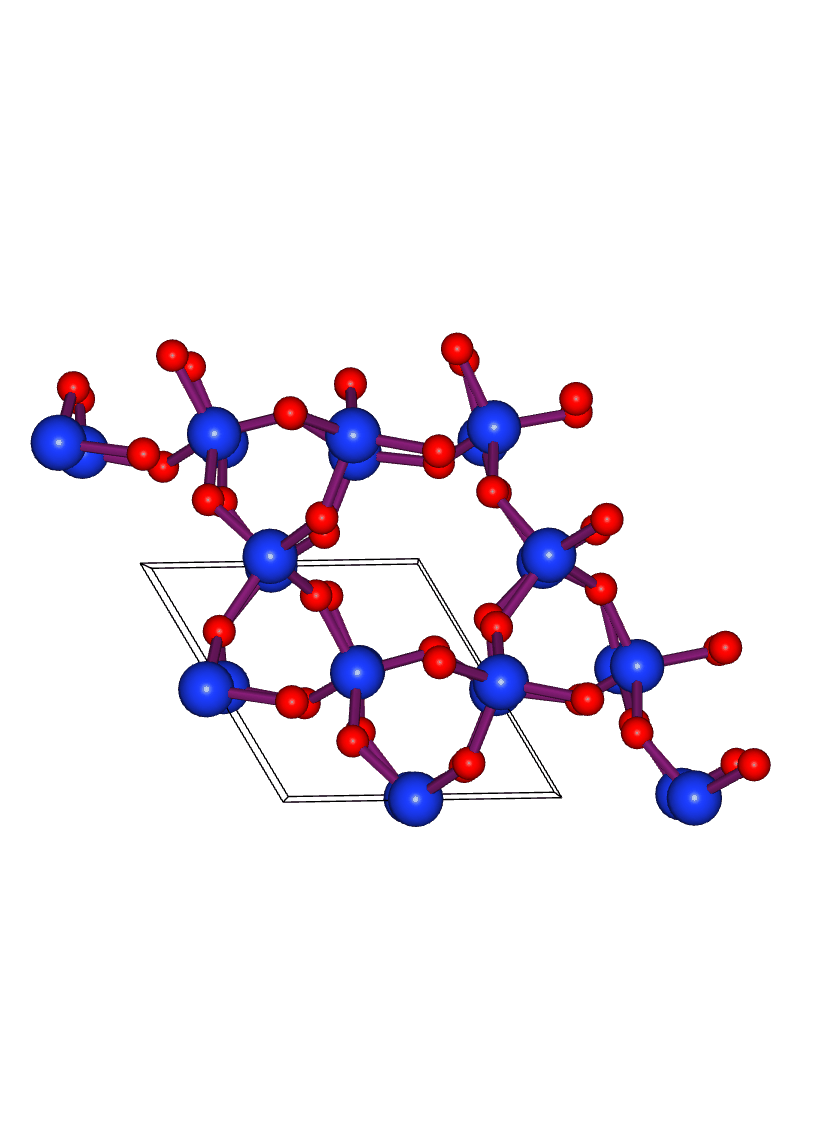

Supplement: Supplementary file 3 — Supplementary Video 1 from Fig. 5c visualizing mode X at Q1 shows that it involves a circular motion of the atoms. [file 41586_2023_6016_MOESM3_ESM.gif]

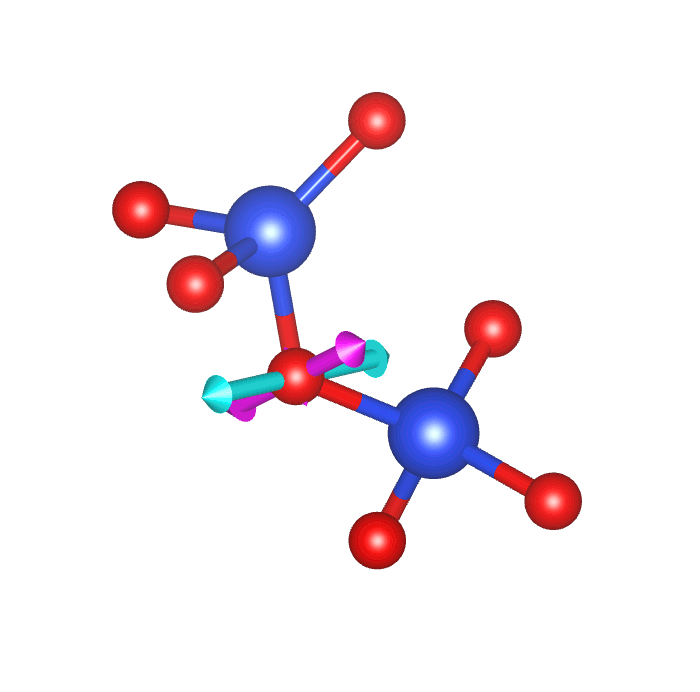

Supplement: Supplementary file 4 — Supplementary Video 2 visualizes the evolution of the local charge quadrupoles at the O site when the chiral phonon mode is excited. [file 41586_2023_6016_MOESM4_ESM.gif]
